# Supplementary material for: Glia inflammation and cell death pathways drive disease progression in preclinical and early AD
Source: EMBO Mol Med. 2025 Oct 10;17(11):3064–79. doi: 10.1038/s44321-025-00316-1 (PMC12603189; doi:10.1038/s44321-025-00316-1)
Supplement: Supplementary file 1 — Appendix [file 44321_2025_316_MOESM1_ESM.pdf]

APPENDIX MATERIALS

Table of contents:

Appendix Fig. S1: Abnormal pathways across the AD continuum. Page 2
Appendix Fig. S2: Neuronal dysfunction cluster is associated with AD hallmarks. Page 3
Appendix Fig. S3: Pathophysiological pathways are active in early disease stages. Page 4
Appendix Fig. S4: Synaptic signaling is associated with AD hallmarks. Page 6
Appendix Fig. S5: Association of disease pathways with progression in CI. Page 8
Appendix Fig. S6: Neuronal pathologies do not mediate the effect of neuroinflammation on glia activation. Page 9
Appendix Table S1: Patient demographics separated by diagnostic groups in TRIAD. Page 10
Appendix Table S2: Patient demographics separated by diagnostic groups in ADNI. Page 11
Appendix Table S3: Top gene ontology terms. Page 12
Appendix Table S4: Defining proteins of biological pathways. Page 13
Appendix Table S5: Patient demographics separated by A/T/N in ADNI. Page 14
Appendix Table S6: Patient demographics separated by A/T/N in TRIAD. Page 15

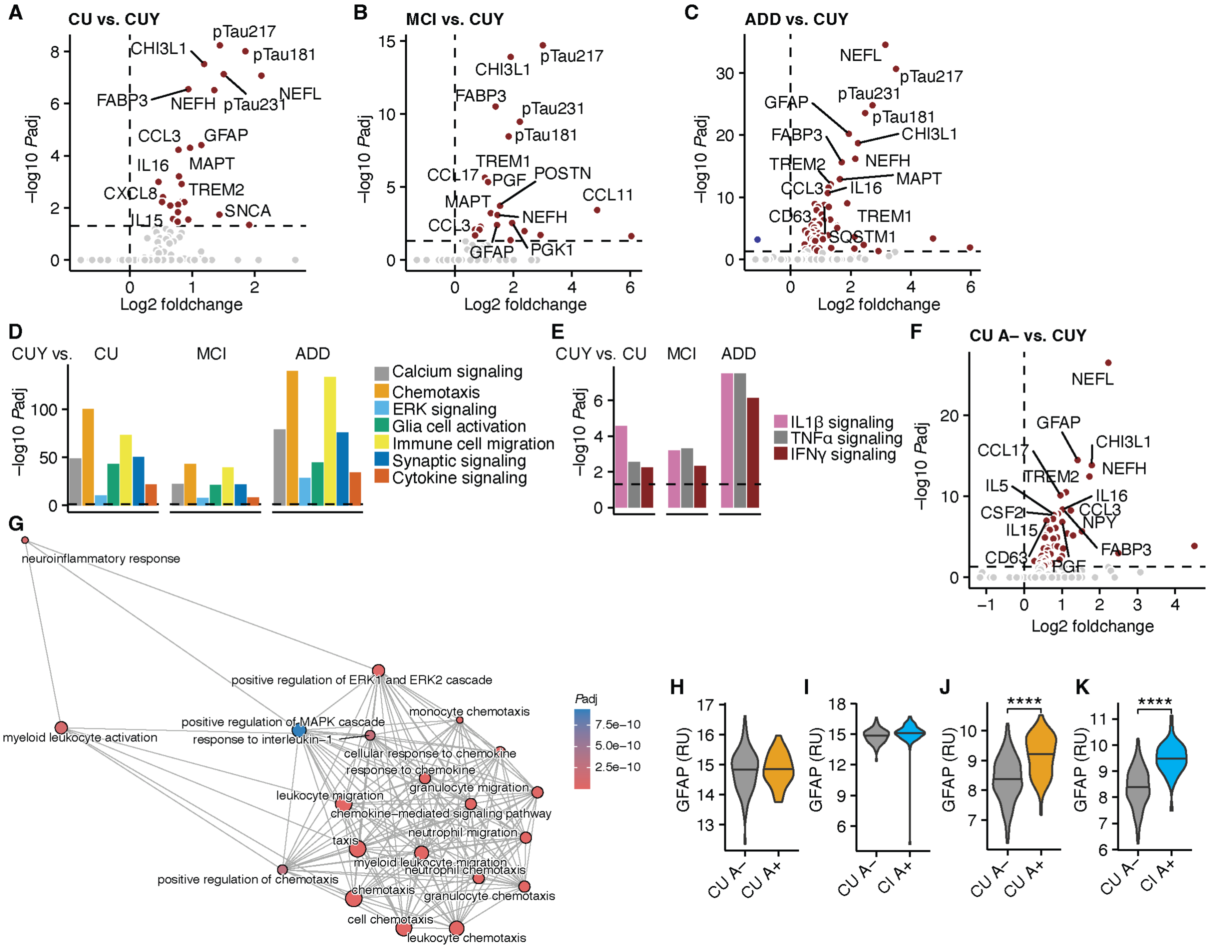

Appendix Fig. S1: Abnormal pathways across the AD continuum. (A-C) Volcano plots of CSF proteins in cognitively unimpaired older than 30 years (CU, n = 154; A), mild cognitive impairment (MCI, n = 39; B), and Alzheimer's disease dementia (ADD, n = 50; C) in comparison to cognitively unimpaired younger than 30 years (CUY, n = 32). Differentially abundant proteins are labeled with colors. (D and E) Negative log10 adjusted *P*-value of biological process gene ontology (GO) analyses of the indicated GO terms of the differentially more abundant proteins in CU, MCI and AD vs. CUY. (F) Volcano plots of CSF proteins in cognitively unimpaired older than 30 years with negative Aβ status (CU A-, *n* = 160; A) and cognitively unimpaired younger than 30 years (CUY, *n* = 32). (G) Emapplot of the differentially more abundant CSF proteins in CU A- (*n* = 160) vs. CUY A- (*n* = 32). (H and I) Log2-transformed CSF GFAP NULISA values of CU A- vs. CU A+ (H), and CU A- vs. CI A+ (I). (J and K) Log2-transformed plasma GFAP NULISA values of CU A- vs. CU A+ (J), and CU A- vs. CI A+ (K). If not stated otherwise, two-tailed *t*-test with FDR-correction were used for group comparisons. ****$P < 0.0001$.

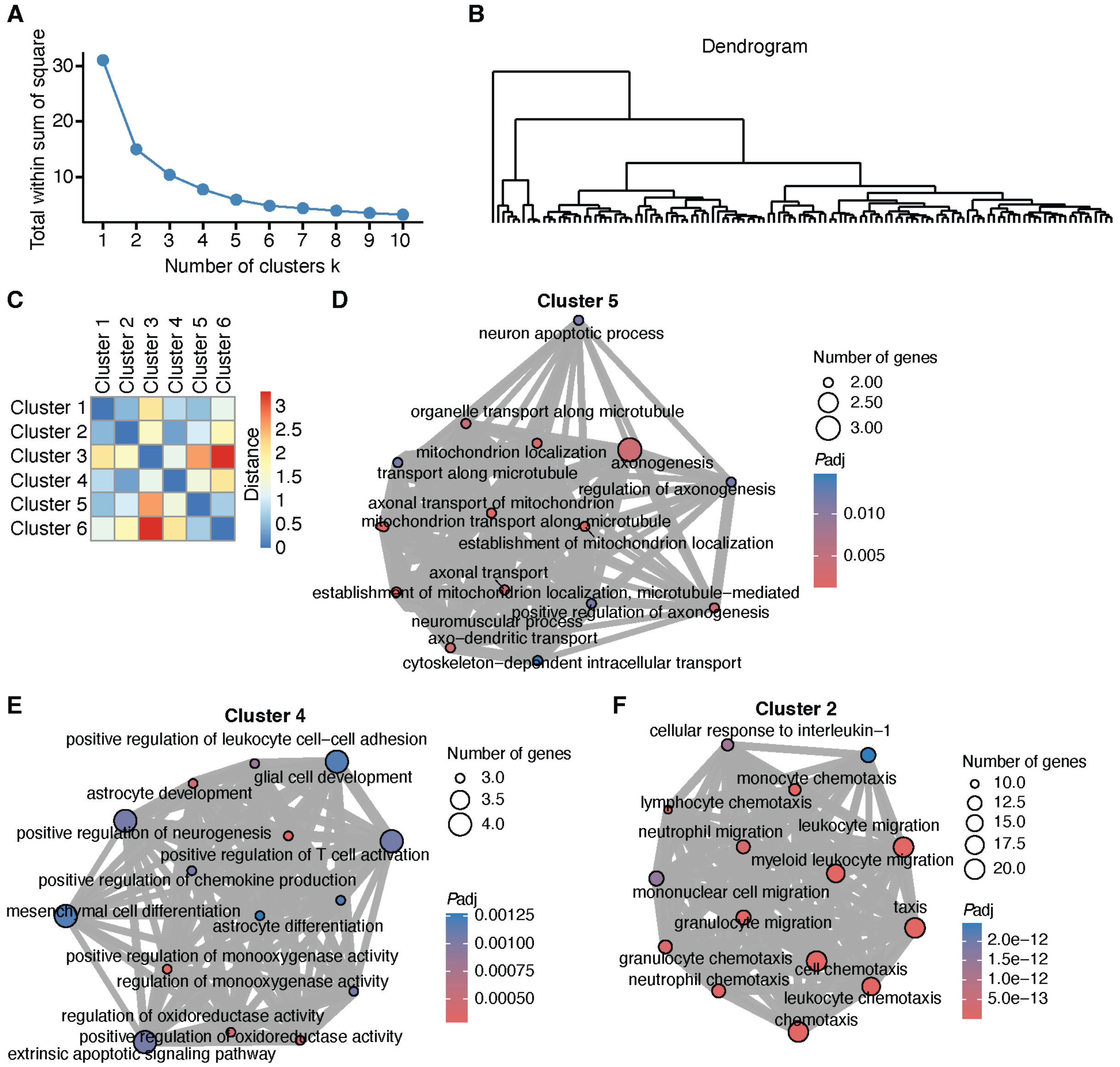

Appendix Fig. S2: Neuronal dysfunction cluster is associated with AD hallmarks. (A and B) Elbow plot (A) and dendrogram plot (B) of hierarchical clustering analysis of Spearman correlation coefficients of all measured proteins with hippocampal volume, neocortical [^{18}F]AZD4694 SUVR, [^{18}F]MK6240 SUVR in Braak I-VI and meta-ROI, Braak stages, mini-mental state examination (MMSE), clinical dementia rating (CDR) and CDR-SOB. (C) Similarity matrix of all identified clusters. (D) Gene ontology biological process analysis of all proteins that were included in cluster 5. (E) Gene ontology biological process analysis of all proteins that were included in cluster 4. (F) Gene ontology biological process analysis of all proteins that were included in cluster 2.

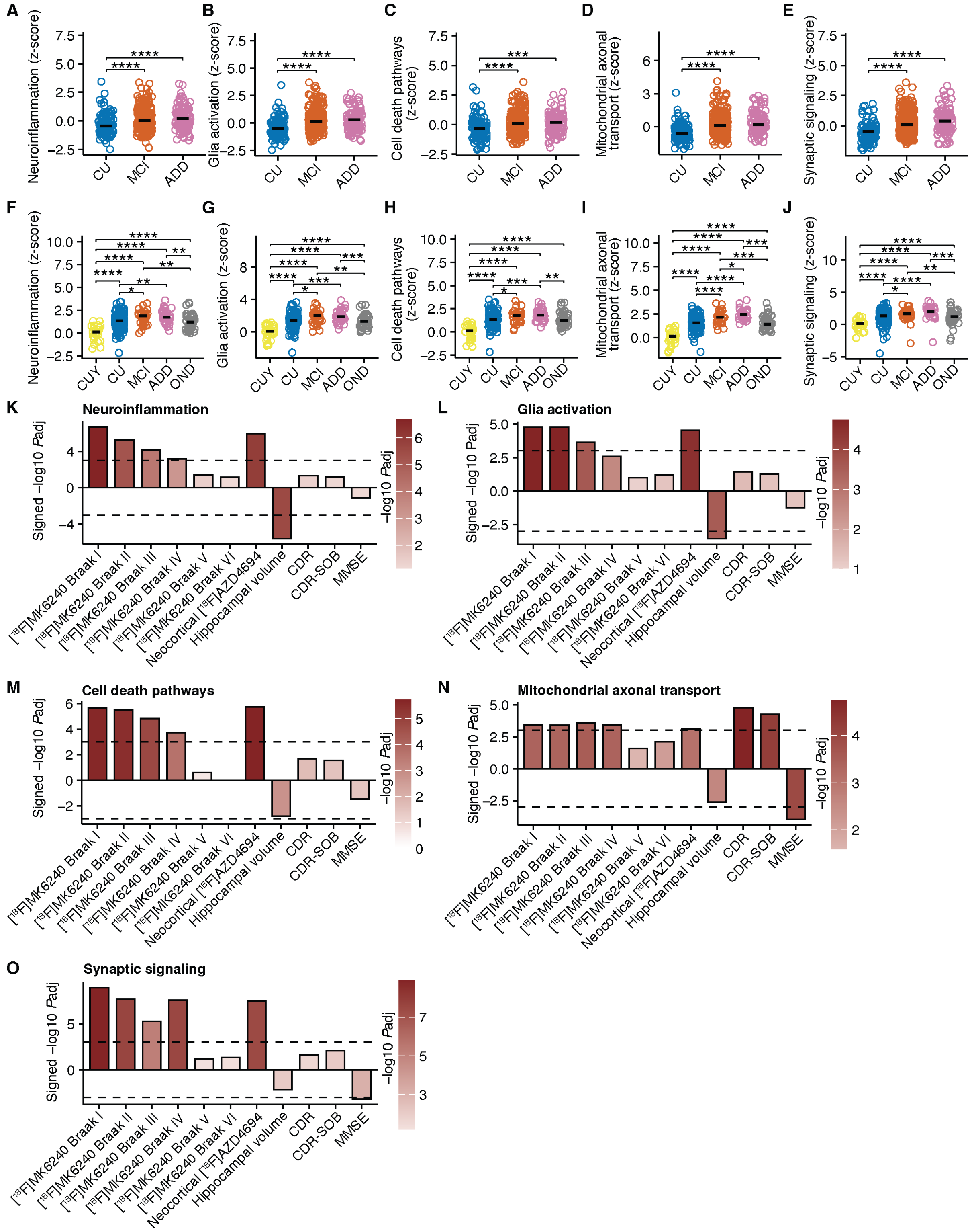

Appendix Fig. S3: Pathophysiological pathways are active in early disease stages. (A-E) Z-score of biological pathways "Neuroinflammation" (A; ADD vs. CU, P = 2.3 × 10^{-5}; ADD vs. MCI, P = 0.57; CU vs. MCI, P = 1.1 × 10^{-5}), "Glia activation" (B; ADD vs. CU, P = 1.5 × 10^{-8}; ADD vs. MCI, P = 0.98; CU vs. MCI, P = 2.5 × 10^{-11}), "Cell death pathways" (C; ADD vs. CU, P = 0.00012; ADD vs. MCI, P = 0.61; CU vs. MCI, P = 1.4 × 10^{-6}), "Mitochondrial axonal transport" (D; ADD vs. CU, P = 2.8 × 10^{-11}; ADD vs. MCI, P = 0.57; CU vs. MCI, P = 4.2 × 10^{-13}), and "Synaptic Signaling" (E; ADD vs. CU, P = 1.1 × 10^{-12}; ADD vs. MCI, P = 0.25; CU vs. MCI, P = 4.7 × 10^{-14}) in cognitively unimpaired older than 30 years (CU, n = 114), mild

cognitive impairment (MCI, n = 213), and patients with Alzheimer's disease dementia (ADD, n = 103) in ADNI. (F-J) Z-score of biological pathways "Neuroinflammation" (F; CUY vs. CU, $P = 1.7 \times 10^{-9}$; CUY vs. MCI, $P = 2.2 \times 10^{-10}$; CUY vs. ADD, $P = 1.8 \times 10^{-11}$; CUY vs. OND, $P = 9.1 \times 10^{-8}$; CU vs. MCI, $P = 0.097$; CU vs. ADD, $P = 0.017$; CU vs. OND, $P = 0.58$; MCI vs. ADD, $P = 0.54$; MCI vs. OND, $P = 0.069$; ADD vs. OND, $P = 0.016$), "Glia activation" (G; CUY vs. CU, $P = 1.2 \times 10^{-9}$; CUY vs. MCI, $P = 2.5 \times 10^{-10}$; CUY vs. ADD, $P = 1.3 \times 10^{-11}$; CUY vs. OND, $P = 1.1 \times 10^{-7}$; CU vs. MCI, $P = 0.12$; CU vs. ADD, $P = 0.014$; CU vs. OND, $P = 0.42$; MCI vs. ADD, $P = 0.44$; MCI vs. OND, $P = 0.054$; ADD vs. OND, $P = 0.0074$), "Cell death pathways" (H; CUY vs. CU, $P = 4.2 \times 10^{-11}$; CUY vs. MCI, $P = 1.9 \times 10^{-11}$; CUY vs. ADD, $P = 1.8 \times 10^{-13}$; CUY vs. OND, $P = 1.9 \times 10^{-9}$; CU vs. MCI, $P = 0.18$; CU vs. ADD, $P = 0.0080$; CU vs. OND, $P = 0.62$; MCI vs. ADD, $P = 0.30$; MCI vs. OND, $P = 0.13$; ADD vs. OND, $P = 0.0096$), "Mitochondrial axonal transport" (I; CUY vs. CU, $P = 2.5 \times 10^{-12}$; CUY vs. MCI, $P < 2 \times 10^{-16}$; CUY vs. ADD, $P < 2 \times 10^{-16}$; CUY vs. OND, $P = 1.3 \times 10^{-11}$; CU vs. MCI, $P = 2.4 \times 10^{-5}$; CU vs. ADD, $P = 3.8 \times 10^{-12}$; CU vs. OND, $P = 0.97$; MCI vs. ADD, $P = 0.042$; MCI vs. OND, $P = 0.00032$; ADD vs. OND, $P = 2.4 \times 10^{-8}$), and "Synaptic Signaling" (J; CUY vs. CU, $P = 6.3 \times 10^{-9}$; CUY vs. MCI, $P = 3.3 \times 10^{-8}$; CUY vs. ADD, $P = 8.2 \times 10^{-11}$; CUY vs. OND, $P = 1.2 \times 10^{-5}$; CU vs. MCI, $P = 0.19$; CU vs. ADD, $P = 0.0058$; CU vs. OND, $P = 0.38$; MCI vs. ADD, $P = 0.25$; MCI vs. OND, $P = 0.080$; ADD vs. OND, $P = 0.0033$) in cognitively unimpaired younger than 30 years (CUY, n = 32), cognitively unimpaired older than 30 years (CU, n = 154), mild cognitive impairment (MCI, n = 39), Alzheimer's disease dementia (ADD, n = 50) and individuals with other neurological diseases (OND, n = 107) in TRIAD. (K-O) Signed -log10 of the adjusted *P*-value of individual scores for "Neuroinflammation" (K), "Glia activation" (L), "Cell death pathways" (M), "Mitochondrial axonal transport" (N), and "Synaptic Signalling" (O) with hippocampal volume, neocortical [^{18}F]AZD4694 SUVR, [^{18}F]MK6240 SUVR in Braak I-VI and meta-ROI, Braak stages, mini-mental state examination (MMSE), clinical dementia rating (CDR) and CDR-SOB in TRIAD. Dashed lines show a -log10 of 0.01.

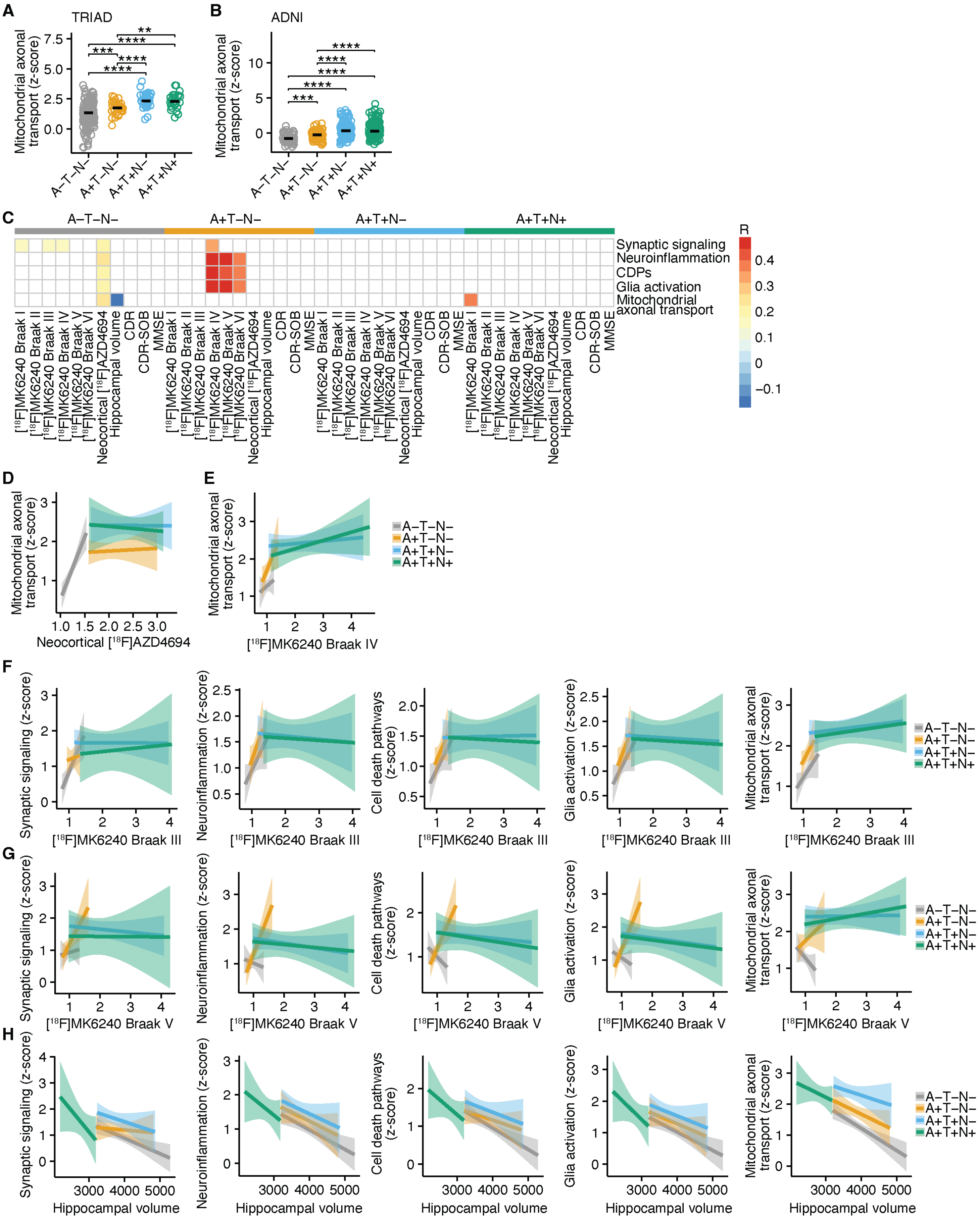

Appendix Fig. S4: Synaptic signaling is associated with AD hallmarks. (A) Z-score of the biological pathway "Mitochondrial axonal transport" in A-T-N- (n = 171), A+T-N- (n = 33), A+T+N- (n = 35), and A+T+N+ (n = 25) in TRIAD (A-T-N- vs. A+T-N-, $P = 0.00014$; A-T-N- vs. A+T+N-, $P = 4.4 \times 10^{-13}$; A-T-N- vs. A+T+N+, $P = 2.9 \times 10^{-8}$; A+T-N- vs. A+T+N-, $P = 2.1 \times 10^{-5}$; A+T-N- vs. A+T+N+, $P = 0.0012$; A+T+N- vs. A+T+N+, $P = 0.64$). Unpaired *t*-test with FDR-correction was used for statistical comparisons. (B) Z-score of the biological pathway "Mitochondrial axonal transport" in A-T-N- (n = 68), A+T-N- (n = 77), A+T+N- (n = 145), and A+T+N+ (n = 140) in ADNI (A+T+N+ vs. A-T-N-, $P = 1.4 \times 10^{-19}$; A+T+N+ vs. A+T-N-, $P = 2.0$

$\times 10^{-10}$; A+T+N+ vs. A+T+N-, $P = 0.94$; A-T-N- vs. A+T-N-, $P = 0.00019$; A-T-N- vs. A+T+N-, $P = 6.7 \times 10^{-21}$; A+T-N- vs. A+T+N-, $P = 4.2 \times 10^{-11}$). Unpaired *t*-test with FDR-correction was used for statistical comparisons. (C) Spearman correlation coefficients separated by A/T/N in individuals older than 50 years (n = 145) of the biological pathways “Neuroinflammation”, “Glia activation”, “Mitochondrial axonal transport”, “Cell death pathways” (CDPs), and synaptic signaling with hippocampal volume, neocortical [^{18}F]AZD4694 SUVR, [^{18}F]MK6240 SUVR in Braak I-VI and meta-ROI, Braak stages, mini-mental state examination (MMSE), clinical dementia rating (CDR) and CDR-SOB in TRIAD. White tiles represent *P*-value > 0.05. (D and E) Regression lines and 95% confidence intervals of the association between “Synaptic signaling” and neocortical [^{18}F]AZD4694 SUVR (D) and Braak IV [^{18}F]MK6240 SUVR (E) separated by A/T/N in TRIAD. Significance and correlation coefficients are shown in Fig. 4I. (F-H) Regression lines and 95% confidence intervals of the association between “Synaptic signaling”, “Neuroinflammation”, “Glia activation”, “Mitochondrial axonal transport”, and “Cell death pathways” with Braak III [^{18}F]MK6240 SUVR (F), Braak V [^{18}F]MK6240 SUVR (G), and hippocampal volume (H) separated by A/T/N in TRIAD. Significance and correlation coefficients are shown in Fig. 4I. *$P < 0.05$, **$P < 0.01$, ****$P < 0.0001$.

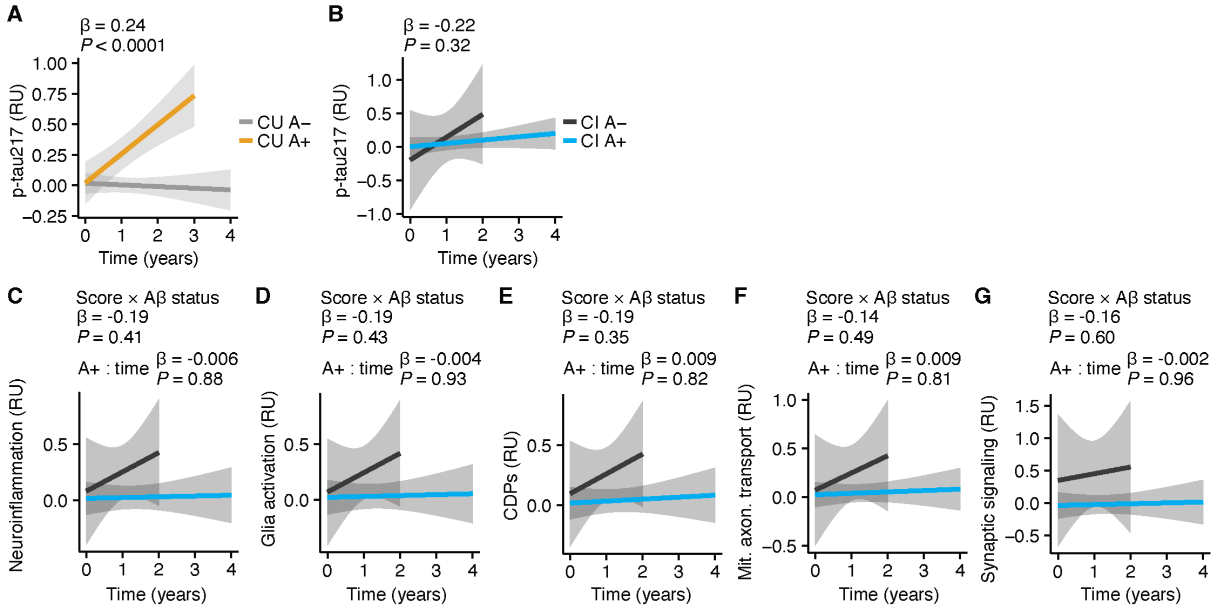

Appendix Fig. S5: Association of disease pathways with progression in CI. (A) Longitudinal change of CSF p-tau217 in cognitively unimpaired with PET proven Aβ accumulation (CU A+, n = 22), and without Aβ accumulation (CU A-, n = 84). The data was normalized to the first visit. The interaction between p-tau217 and amyloid status over time (years) was tested using mixed linear models. (B) Longitudinal change of CSF p-tau217 in cognitively impaired with PET proven Aβ accumulation (CI A+, n = 4), and without Aβ accumulation (CI A-, n = 36). The data was normalized to the first visit. The interaction between p-tau217 and amyloid status over time (years), and the longitudinal increase in A+ participants was tested using mixed linear models. The increase in A- participants could not be tested because of a insufficient number of longitudinal observations (C-G) Longitudinal change of CSF signatures for “Neuroinflammation” (C), “Glia activation” (D), “Cell death pathways” (CDPs) (E), “Mitochondrial axonal transport” (F), and “Synaptic signalling” (G) in cognitively unimpaired with PET proven Aβ accumulation (CI A+, n = 4), and without Aβ accumulation (CI A-, n = 36). The data was normalized to the first visit. The interaction between the signatures and amyloid status over time (years) was tested using mixed linear models. The β-values and *P*-values are shown in the figure.

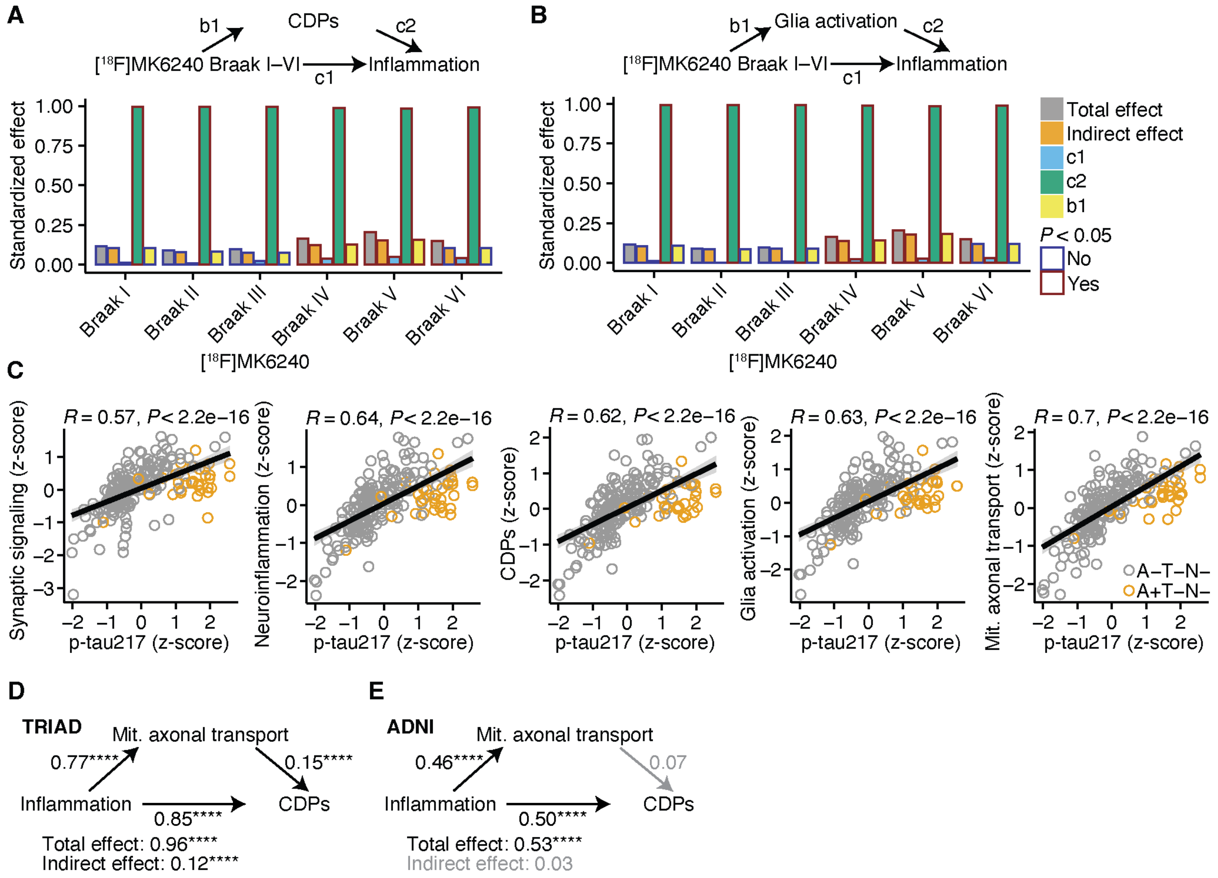

Appendix Fig. S6: Neuronal pathologies do not mediate the effect of neuroinflammation on glia activation. (A and B) Mediation analysis in A+T+ (n = 60) using [18F]MK6240 SUVR in Braak I-VI as predictors, neuroinflammation as predicted variable, and cell death pathways (CDPs; A) and glia activation (B) as mediators. The standardized effects for all paths as well as the total and indirect effects are shown. Significance is indicated as coloring. (C) Correlation analysis of p-tau217 with the signatures for "Synaptic signaling", "Neuroinflammation", "Cell death pathways" (CDPs), "Glia activation", and "Mitochondrial axonal transport" in A-T-N- (grey) and A+T-N- (orange). Spearman correlation analysis was performed. Significance and correlation coefficients are shown in the figure. (D and E) Mediation analysis in A-T- and A+T- in TRIAD (D, n = 204) and ADNI (E, n = 145) using neuroinflammation as predictor, activation of CDPs as predicted variable, and and mitochondrial axonal transport as mediator. The standardized effects for all paths as well as the total and indirect effects are shown. ****P < 0.0001.

Appendix Table S1: Patient demographics separated by diagnostic groups in TRIAD.

	CUY	CU	MCI	ADD	OND
N total , female (%)	32 (62.5)	154 (64.29)	39 (51.28)	50 (38)	107 (58.88)
APOE ε4 carriership (%)	6 (0.19)	32 (0.21)	17 (0.44)	26 (0.52)	15 (0.14)
Age, mean (sd)	22.66 (1.99)	68.25 (8.27)	71.21 (5.4)	65.81 (7.86)	68.22 (9.15)
Education, years, mean (sd)	16.73 (1.52)	15.41 (3.46)	16.38 (4.17)	14.59 (3.32)	14.78 (3.56)
Hippocampal volume, mm^3, mean (sd)	4366.15 (524.01)	3823.5 (419.89)	3451.18 (476.25)	3275.87 (545.42)	3570.56 (505.41)
Neocortical [^{18}F]AZD4694, SUVR, mean (sd)	1.17 (0.07)	1.4 (0.36)	2.37 (0.47)	2.24 (0.52)	1.24 (0.12)
Meta-ROI [^{18}F]MK6249, SUVR, mean (sd)	0.93 (0.11)	0.96 (0.15)	1.43 (0.51)	2.4 (0.87)	1 (0.38)

Abbreviations: CUY = Cognitively unimpaired young, CU = Cognitively unimpaired, MCI = Mild Cognitive Impairment, ADD = Alzheimer's disease dementia, OND = Other neurological disease, SUVR = Standard uptake ratio value, ROI = Region of interest

Appendix Table S2: Patient demographics separated by diagnostic groups in ADNI.

	CU	MCI	ADD
N total, female (%)	114 (43.86)	213 (41.78)	103 (40.78)
APOE ε4 carriership (%)	30 (26.32)	148 (69.48)	83 (80.58)
Age, mean (sd)	74.5 (5.84)	73.11 (7.17)	74.57 (8.52)
Education, years, mean (sd)	16.26 (2.79)	15.94 (2.93)	15.32 (2.82)
Hippocampal volume, mm^3, mean (sd)	3744.73 (354.63)	3320.4 (529.85)	2944.6 (465.14)
Neocortical [^{18}F]Florbetapir, SUVR, mean (sd)	1.12 (0.17)	1.39 (0.18)	1.47 (0.16)
CSF Aβ42, pg/mL, mean (sd)	1145.47 (420.31)	720.99 (247.05)	576.36 (200.12)
CSF p-tau181, pg/mL, mean (sd)	20.48 (9.12)	35.98 (16.57)	38.91 (14.24)

Abbreviations: CU = Cognitively unimpaired, MCI = Mild Cognitive Impairment, ADD = Alzheimer's disease dementia, SUVR = Standard uptake ratio value

Appendix Table S3: Top gene ontology terms.

TRIAD: CU A- vs CUY A-	TRIAD: CI A+ vs CU A-	ADNI: CI A+ vs CU A-	ADNI: CU A+ vs CU A-
cell chemotaxis	positive regulation of lipid metabolic process	axonogenesis	axonogenesis
leukocyte migration	negative regulation of protein localization	axon development	axon guidance
leukocyte chemotaxis	positive regulation of phospholipid biosynthetic process	neuron projection morphogenesis	neuron projection guidance
chemotaxis	regulation of phosphatidylcholine metabolic process	neuron projection development	regulation of neuron projection development
taxis	regulation of lipid metabolic process	plasma membrane bounded cell projection morphogenesis	chemotaxis
chemokine-mediated signaling pathway	internal protein amino acid acetylation	cell morphogenesis involved in neuron differentiation	taxis
myeloid leukocyte migration	negative regulation of mitochondrial membrane potential	neuron development	synapse organization
response to chemokine	negative regulation of membrane potential	regulation of neuron projection development	regulation of axonogenesis
cellular response to chemokine	Golgi localization	cellular component morphogenesis	synapse assembly
neutrophil chemotaxis	regulation of long-term synaptic depression	regulation of plasma membrane bounded cell projection organization	regulation of chemotaxis
granulocyte chemotaxis	positive regulation of phospholipid metabolic process	regulation of cell projection organization	positive regulation of nervous system development
neutrophil migration	organelle inheritance	cell morphogenesis	regulation of nervous system development
monocyte chemotaxis	Golgi inheritance	synapse organization	membrane fusion
granulocyte migration	phospholipid homeostasis	axon guidance	positive regulation of axonogenesis
positive regulation of ERK1 and ERK2 cascade	regulation of phospholipid biosynthetic process	neuron projection guidance	protein folding
neuroinflammatory response	regulation of cellular response to heat	synapse assembly	Metabolic regulation
myeloid leukocyte activation	NADH regeneration	cell junction organization	cell junction assembly
response to interleukin-1	axonal transport of mitochondrion	synaptic signaling	neuron recognition
positive regulation of chemotaxis	positive regulation of superoxide anion generation	Metabolic regulation	regulation of neurogenesis
positive regulation of MAPK cascade	long-chain fatty acid import into cell	axonogenesis	negative regulation of chemotaxis

Appendix Table S4: Defining proteins of biological pathways.

Synaptic signalling	Neuroinflammation	Cell death pathways	Glia activation	Mitochondrial axonal transport
ACHE	CCL3	BACE1	CCL3	MAPT
APOE	IGF1	CCL3	MAPT	NEFL
BACE1	IL18	NEFL	PSEN1	SOD1
CALB2	MAPT	PARK7	SNCA	UCHL1
GFAP	PSEN1	PSEN1	TREM2	
MAPT	SNCA	SNCA	CX3CL1	
NEFH	TREM2	SNCB	TNF	
NEFL	CX3CL1	SOD1	GFAP	
PSEN1	TNF	TREM2	APOE	
PTN	GFAP	CCL2		
S100B	APOE	CRH		
SNAP25	CCL2	CX3CL1		
SNCA		KDR		
SQSTM1		TNF		
CRH				

Appendix Table S5: Patient demographics separated by A/T/N in ADNI.

	A-T-N-	A+T-N-	A+T+N-	A+T+N+
N total, female (%)	68 (48.53)	77 (18.18)	145 (41.38)	140 (52.86)
APOE ε4 carriership (%)	8 (11.76)	38 (49.35)	100 (68.97)	115 (82.14)
Age, mean (sd)	73.02 (5.42)	73.15 (6.55)	72.18 (8.09)	76.3 (6.78)
Education, years, mean (sd)	16.18 (2.94)	15.78 (2.97)	16.44 (2.52)	15.2 (3.04)
Hippocampal volume, mm^3, mean (sd)	3818.36 (337.3)	3646.39 (327.85)	3540.48 (355.25)	2678.84 (264.4)
Neocortical [^{18}F]Florbetapir, SUVR, mean (sd)	1.01 (0.05)	1.3 (0.15)	1.42 (0.18)	1.45 (0.17)
CSF Aβ42, pg/mL, mean (sd)	1384.68 (297.49)	707.56 (317.6)	733.71 (233.21)	632.08 (226.93)
CSF p-tau181, pg/mL, mean (sd)	16.14 (3.52)	19.06 (5.01)	39.69 (13.26)	40.61 (15.79)

Abbreviations: SUVR = Standard uptake ratio value, ROI = Region of interest

Appendix Table S6: Patient demographics separated by A/T/N in TRIAD.

	A-T-N-	A+T-N-	A+T+N-	A+T+N+
N total, female (%)	171 (59.06)	33 (57.58)	35 (40)	25 (64)
APOE ε4 carriership (%)	33 (0.19)	11 (0.33)	21 (0.6)	11 (0.44)
Age, mean (sd)	60.78 (18.39)	68.82 (9.8)	66.94 (8.49)	69.84 (7.87)
Education, years, mean (sd)	15.46 (3.34)	15.24 (4.37)	15.54 (3.58)	15.32 (3.56)
Hippocampal volume, mm^3, mean (sd)	3924.57 (421.84)	3844.05 (337.19)	3672.8 (371.59)	2814.48 (275.63)
Neocortical [^{18}F]AZD4694, SUVR, mean (sd)	1.23 (0.1)	2.05 (0.41)	2.34 (0.38)	2.5 (0.42)
Meta-ROI [^{18}F]MK6249, SUVR, mean (sd)	0.92 (0.09)	0.97 (0.09)	2.03 (0.76)	2.21 (0.76)

Abbreviations: SUVR = Standard uptake ratio value, ROI = Region of interest
